# Treatment with Hyaluronic Acid and Collagen-Polyvinylpyrrolidone Improves Extracellular Matrix Assembly for Scarring after Tracheal Resection

**DOI:** 10.1155/2020/3964518

**Published:** 2020-08-27

**Authors:** J. Raúl Olmos-Zuñiga, Matilde Baltazares-Lipp, Claudia Hernández-Jiménez, Rogelio Jasso-Victoria, Miguel Gaxiola-Gaxiola, Mariana Silva-Martínez, Marco Antonio Iñiguez-García, A. Ivan González-González, Juan Carlos Vázquez-Minero, Antonia Luna-Flores, Norma Solis-Alanis, Mario Enrique Baltazares-Lipp

**Affiliations:** ^1^Lung Transplantation Research Unit, Department of Surgical Research, Instituto Nacional de Enfermedades Respiratorias Ismael Cosio Villegas, Mexico City, Mexico; ^2^Department of Surgical Research, Instituto Nacional de Enfermedades Respiratorias Ismael Cosio Villegas, Mexico City, Mexico; ^3^Laboratory of Morphology, Instituto Nacional de Enfermedades Respiratorias Ismael Cosio Villegas, Mexico City, Mexico; ^4^Subdirection of Surgery, Instituto Nacional de Enfermedades Respiratorias Ismael Cosio Villegas, Mexico City, Mexico; ^5^Department of Otolaryngology-Head and Neck Surgery, Instituto Nacional de Enfermedades Respiratorias Ismael Cosio Villegas, Mexico City, Mexico; ^6^Department of Surgery, Facultad de Medicina Veterinaria y Zootecnia, Universidad Nacional Autónoma de México, Mexico City, Mexico; ^7^External Consultation Service, Instituto Nacional de Enfermedades Respiratorias Ismael Cosio Villegas, Mexico City, Mexico

## Abstract

Treatment of tracheal stenosis is occasionally performed in combination with wound healing modulators to manipulate new extracellular matrix (ECM) formation and prevent fibrosis. Hyaluronic acid (HA) and collagen-polyvinylpyrrolidone (collagen-PVP) decrease fibrosis in experimental tracheal healing. However, they have not been used clinically as their effect on ECM components, which modify tracheal scarring, has not been described. *Objective*. To evaluate the effect of the application of HA, collagen-PVP, a mixture of HA and collagen-PVP (HA+collagen-PVP), and mitomycin C on the expression of decorin, matrix metalloproteinase 1 (MMP1), and MMP9, as well as the type of collagen and deposits formed in the scar after resection and end-to-end anastomosis (REEA) of the cervical trachea using an experimental model. *Materials and Methods*. Thirty dogs underwent REEA of the cervical trachea and were treated with different wound healing modulators: group I (*n* = 6), control; group II (*n* = 6), HA; group III (*n* = 6), collagen-PVP; group IV (*n* = 6), HA+collagen-PVP; and group V (*n* = 6), mitomycin C. The dogs were evaluated clinically and endoscopically for 4 weeks. Subsequently, macroscopic and microscopic changes, expression of ECM proteins, and collagen deposition in tracheal scars were analysed. *Results*. Groups II, III, and IV showed reduced endoscopic, macroscopic, and microscopic inflammation, improved neovascularization, high decorin expression (*p* < 0.01, analysis of variance (ANOVA)), and moderate expression of MMP1 (*p* < 0.003, ANOVA) and type I and III collagen (*p* < 0.05, Kruskal–Wallis). Groups IV and V developed fewer collagen deposits (*p* < 0.001, ANOVA). *Conclusion*. Treatment with HA and collagen-PVP improved post-REEA healing by increasing neovascularization, stimulating the expression of decorin, and regulating the expression of MMP1, as well as type I and III collagen and their deposition.

## 1. Introduction

Posttraumatic tracheal stenosis (TS) and tracheal restenosis are disproportionate fibrotic tracheal wounds that obstruct the lumen and prevent airflow [1]. They are the result of pathological wound healing processes in which chronic inflammation and ischaemic necrosis alter the production of cytokines, growth factors, and matrix metalloproteinases (MMPs). These alterations cause changes in the expression of extracellular matrix (ECM) components, such as decorin, fibronectin, and collagen, which promote fibrosis at the injury site [2–4].

Treatment of TS is performed through endoscopic procedures or resection and end-to-end anastomosis (REEA) of the affected tracheal segment. However, since TS is the result of pathological scarring, treatment is occasionally performed in combination with the application of wound healing modulators (WHMs), such as mitomycin C (MMC), to pharmacologically manipulate the formation of the new ECM and prevent fibrosis. Of note, these treatments are not 100% effective, as they do not prevent restenosis [1, 5]. For this reason, it is necessary to evaluate alternative WHMs.

Hyaluronic acid (HA) is a fibrogenesis modulator during the foetal healing process. It accelerates the deposition of collagen in the wound and organizes it in a unidirectional and fibrillary pattern similar to normal, to avoid the formation of fibrosis [6].

Collagen-polyvinylpyrrolidone (collagen-PVP) is a drug formed by porcine collagen, treated with gamma rays and PVP, which has fibrinolytic activity. It inhibits the expression of adhesion molecules (endothelial adhesion molecule 1 (ELAM1), vascular cell adhesion molecule 1 (VCAM1)), as well as some proinflammatory and fibrogenic cytokines (interleukin 1*β* (IL1*β*), tumour necrosis factor *α* (TNF*α*), transforming growth factor *β*1 (TGF*β*1), and platelet-derived growth factor (PDGF)) [7].

At Instituto Nacional de Enfermedades Respiratorias Ismael Cosio Villegas (INERICV; Mexico City, Mexico), we have been working with HA and collagen-PVP in experimental models of tracheal healing. We observed that their application at the injured trachea is safe and promotes healing with reduced inflammation, collagen deposits, and fibrosis, as well as absence of stenosis [6, 8]. However, thus far, there are no research studies investigating whether this adequate healing process is due to the regulation provided by these drugs over the expression of the ECM components that produce fibrotic scars. Such research could assist in understanding the reasons these WHMs improve scarring in this organ and provide another potential clinical treatment option.

The aim of this investigation was to evaluate the effect of the application of HA, collagen-PVP, a mixture of HA and collagen-PVP (HA+collagen-PVP), and MMC on the expression of decorin, MMP1, and MMP9 and the type of collagen and deposits formed in the scar after resection and end-to-end anastomosis of the cervical trachea (CT) using an experimental model.

## 2. Materials and Methods

### 2.1. Experimental Animals

Thirty healthy mongrel dogs of either sex, weighing 15–25 kg, were used. The dogs were provided by the INERICV animal house. The protocol of this study was reviewed and approved by the INERICV Bioethics Committee (permit number B14-05). The present research was performed according to the technical specifications for the care and use of laboratory animals of the Official Mexican Standard (NOM-062-ZOO-1999) [9] and the Guide for the Care and Use of Laboratory Animals of the USA [10]. The sample size was established in agreement with the principles of experimental techniques proposed by Balls [11] and Kilkenny et al. [12]. All procedures were performed under general anaesthesia, and all efforts were made to minimize animal suffering.

#### 2.1.1. Study Groups

The animals were randomly assigned into one of five study groups, and a conventional REEA of three CT rings (3rd–5th) was performed, combined with the application of the WHM as follows:

*Group I* (n = 6): REEA of CT rings and treatment with 3 ml of saline solution (control).

*Group II* (n = 6): REEA of CT rings and treatment with 15 *μ*g of HA (hyaluronic acid potassium salt; Fluka Laboratory Chemicals, Riedel-de Häen, Germany) contained in 3 ml of distilled water (dose used in intestinal and tracheal healing experimental trials) [6, 13].

*Group III* (n = 6): REEA of CT rings and treatment with 2.5 mg of collagen-PVP (Fibroquel; Aspid S. A de C.V., Mexico City, Mexico) (dose clinically used for the treatment of skin and tendon problems and used in chronic TS trials) contained in 3 ml of distilled water [6–8, 14].

*Group IV* (n = 6): REEA of CT rings and treatment with a mixture of HA+collagen-PVP at the aforementioned doses.

*Group V* (n = 6): REEA of CT rings and treatment with 1.2 mg of MMC (Mixandex; Pisa, S.A. de C.V., Guadalajara, Mexico) (dose used for the clinical treatment of TS) contained in 3 ml of distilled water [5].

In group I, normal saline, widely used to clean wounds, was used as its isotonic nature does not interfere with the healing process [15]. Moreover, it is a pharmacologically inert substance commonly used as a control in clinical trials. Distilled water was used as an excipient to all drugs because it is used as an excipient in the production of parenteral and other preparations, for lavage to check homeostasis at the end of surgery and for wound cleansing. Moreover, its pH is similar to that of the normal skin of healthy individuals (pH 5.4–5.9) [15, 16]. Notably, the pH of distilled water does not affect the pH of the collagen-PVP, since the drug has a citrate buffer which stabilizes the pH. Moreover, the association of both drugs confers various physicochemical properties, such as the impossibility of forming a gel when diluted in culture medium at 37°C and neutral pH [14].

### 2.2. Experimental Model

#### 2.2.1. Anaesthesia and Surgical Procedure

Anaesthesia was induced with an intravenous injection of xylazine hydrochloride (0.1 mg/kg) (Rompun; Bayer, Leverkusen, Germany) and propofol (6 mg/kg) (Diprivan; AstraZeneca, Edo. Mexico, Mexico), followed by endotracheal intubation (endotracheal tube; Rusch, Kamunting, Malaysia). General anaesthesia was maintained with 1.5% isoflurane (Forane; Abbott Mexico S.A. de C.V., Mexico City, Mexico).

The anaesthetized dog was placed in the dorsal decubitus position, and a midline incision in the ventral cervical region was performed, followed by dissection until the trachea was reached. All tracheal circumferences were dissected, and three rings were resected (3rd–5th rings). Subsequently, end-to-end tracheal anastomosis was conducted with 3-0 poliglactine 910 (Vicryl; Ethicon, NJ, USA), with continuous sutures in the membranous portion and a simple interrupted suture pattern in the cartilaginous portion. Finally, conventional closure was performed. The resected rings were collected and used as control samples.

All animals received postoperative enrofloxacin (5 mg/kg, IM) (Baytril; Bayer, Leverkusen, Germany) for 5 days as an antibiotic and flunixin meglumine (0.1 mg/kg, IM) (Napzin; Pisa Agropecuaria, Hidalgo, Mexico) as an analgesic. The animals were allowed to recover in their cages, where they remained for 4 weeks postsurgery. All surgical procedures were performed by the same surgical team.

#### 2.2.2. Application of Treatment

During the surgical procedure, all animals received treatment on both ends of the resected trachea. Subsequently, in groups I, II, III, and IV, the treatment was applied via tracheoscopy topical instillation on a weekly basis for 4 weeks. In group V, MMC was applied only once (during the surgical process).

### 2.3. Evaluation

For 4 weeks postsurgery, the animals were evaluated as follows:

#### 2.3.1. Clinical Evaluation

Clinical evaluation was performed daily during the first postoperative week, every third day during the second week, and weekly for the remaining 2 weeks. The purpose of the evaluation was to detect the presence of dyspnoea and stridor.

#### 2.3.2. Endoscopic Evaluation

Under general anaesthesia, endoscopic tracheal evaluation was performed presurgery, immediately post-REEA, and on a weekly basis for the remaining duration of the study, to assess the healing process and the presence of inflammation, infection, fistulas, or TS (using the Myers–Cotton scale) [17].

#### 2.3.3. Macroscopic and Microscopic Evaluation

At the end of the study, all animals were euthanized with an overdose of sodium pentobarbital (100 mg/kg intraperitoneal; Anestesal; Pfizer, S.A. de C.V., Guadalajara, Mexico) [9, 10]. Macroscopic examination was performed to evaluate the intraluminal and extraluminal tracheal healing, degree of stenosis, fibrosis formation, and the presence of dehiscence or infection. At the end of this evaluation, the samples of each group were split into two portions: one for microscopic and immunohistochemical study and the other to assess collagen deposition in the healed tissue.

For histological evaluation, the collected samples were fixed in 10% formaldehyde, embedded in paraffin, and stained with haematoxylin-eosin (for general histology, neovascularization, and the degree of inflammation) and Masson's trichrome for the determination of collagenous tissues (fibrosis, shape, and distribution of collagen fibres). Masson's trichrome staining was performed to visualize collagen fibres as follows. The tissue sections were deparaffinized and hydrated with distilled water. Next, they were refixed in Bouin's, solution for 1 h at 56°C (to improve staining quality), washed for 1–2 min in running tap water at room temperature, rinsed in distilled water, stained with Weigert's Iron Hematoxylin (equal parts of haematoxylin A and haematoxylin B) for 10 min at room temperature, rinsed again in running warm tap water for 10 min, and washed in distilled water. Subsequently, the samples were stained with Biebrich scarlet-acid fuchsin solution for 10–15 min and washed in distilled water. Afterwards, phosphomolybdic acid-phosphotungstic acid solution was added directly on the slides of each sample for 3–4 min at room temperature, transferred directly (without rinsing) to aniline blue solution and stained for 5–10 min, rinsed briefly in distilled water, and differentiated in 1% acetic acid solution for 2–5 min. Immediately after washing in distilled water and dehydration with 95% ethyl alcohol, absolute ethyl alcohol was used to remove Biebrich scarlet-acid fuchsin staining, and the slides were cleared with xylene. Finally, the samples were mounted with resinous mounting medium and evaluated [18].

A double-blind analysis was performed using light microscopy. The assessment was conducted in the entire circumference of the sample with a semiquantitative scale described by Veiga et al. [19]. A grade was assigned to each parameter evaluated according to the intensity (grade 1: absent 0–10%; grade 2: mild 11–25%; grade 3: moderate 26–50%; and grade 4: severe 51–100%).

#### 2.3.4. Immunohistochemical Evaluation

The expression of ECM components in the tracheal scar was determined using immunohistochemistry (IHC) in tracheal tissue sections with antidecorin (1 : 250 dilution; ab67449; Abcam, Cambridge, MA, USA), anti-MMP1 (1 : 200 dilution; ab38929; Abcam Cambridge, MA, USA), and anti-MMP9 (1 : 150 dilution; ab38898; Abcam, Cambridge, MA, USA); collagen type I alpha 1 chain (COL1A1; 1 : 50 dilution; D-13 sc-25974; Santa Cruz Biotechnology, Santa Cruz, CA, USA), collagen type II alpha 1 chain (COL2A1; 1 : 200 dilution; sc-52658; Santa Cruz Biotechnology), and collagen type III (1 : 50 dilution; MAB3392, Chemicon International, CA, USA) primary antibodies; and the biotin-streptavidin-peroxidase system (Vectastain Universal Quick Kit; Vector Laboratories, Burlingame, CA, USA). The sections stained for decorin and MMPs were incubated with 3,3′-diaminobenzidine (DAB; Biocare Medical, CA, USA), while those stained for collagen were incubated with aminoethyl carbazole (AEC Substrate Pack; BioGenex, CA, USA). All samples were counterstained with CAT haematoxylin (Biocare Medical). After completing the staining, the samples were visualized with DAB, and the expression was quantified in the entire sample circumference using the ImageJ software (http://rsbweb.nih.gov/ij/) developed by the National Institutes of Health (Bethesda, MD, USA) and the IHC Profiler plugin [20]. The collagen samples were evaluated using the scale described by Choudhury et al. [21] as no plugin can quantify the pixels in the same manner as with the DAB chromogen. Negative and positive controls were included in all evaluations. In all cases, the negative control was a sample of tracheal tissue incubated with a Tris-buffered saline with Tween 20 instead of the primary antibody, which was processed with the same procedure as the other specimens studied. The tissue used as a positive control for the different ECM components was suggested by the data sheet of primary antibodies: decorin: human colon; MMP1: rat vascular endothelium; MMP9: canine lung; collagen types I and III: human placenta; and collagen type II: canine matrix producing carcinoma of the breast.

#### 2.3.5. Biochemical Evaluation of Collagen Deposition

Collagen deposition (collagen/tracheal tissue, mg) in the membranous portions of the resected tracheal rings (presurgery) and the healed anastomosis were biochemically evaluated according to the Woessner method [22]. This approach determines the concentration of hydroxyproline per gram of tracheal tissue. The samples were weighed and placed in an oven at 80°C until the dry weight was obtained and hydrolysed in 1.5 ml of 6 N hydrochloric acid for 36 h. Subsequently, the samples were filtered and placed in an evaporator until the excess acid was removed. In the obtained residue, the pH was adjusted to 7.0, and each sample was reconstituted with distilled water to 10 ml. From this last dilution, an aliquot of 100 *μ*l was obtained to determine the concentration of hydroxyproline. For the evaluation of hydroxyproline, a standard curve was prepared with a solution containing 1 mg of hydroxyproline/1 ml of water and adjusted for the hydroxyproline standards at concentrations of 200, 400, 600, 800, and 1000 *μ*l of hydroxyproline; a blank containing distilled water was included. Subsequently, the following were added in all tubes: 1 ml of 0.05 M chloramine T dissolved in methyl cellosolve, water, and citrate buffer (pH 6.0); 1 ml of 3.0 N perchloric acid; and 1 ml of a solution of 20% paradimethylaminobenzaldehyde in methyl cellosolve. Next, the samples were incubated for 20 min at 60°C, and the absorbance of each sample was detected using a spectrophotometer (DU 640; Beckman Coulter Inc., Brea, CA, USA) at a wavelength of 560 nm. All chemicals were purchased from Sigma-Aldrich (Hydroxyproline Assay Kit; Sigma-Aldrich, St. Louis, MO, USA).

#### 2.3.6. Statistical Analysis

All data were analysed using SPSS version 18.0 for Windows (SPSS Inc., Chicago, IL, USA). All data presented a normal distribution pattern (Kolmogorov–Smirnov test). The statistical analysis of nonparametric data was performed with the Kruskal–Wallis test, whereas parametric data were assessed by analysis of variance (ANOVA) and Dunnett's and Tukey's tests (values were expressed as the mean ± standard deviation); *p* values < 0.05 denoted statistically significant differences.

## 3. Results

All animals survived the surgical procedure and study period. Clinically, only three (50%) subjects in group I showed stridor. None of the studied dogs presented dyspnoea.

### 3.1. Endoscopic and Macroscopic Changes

**Figure 1:**
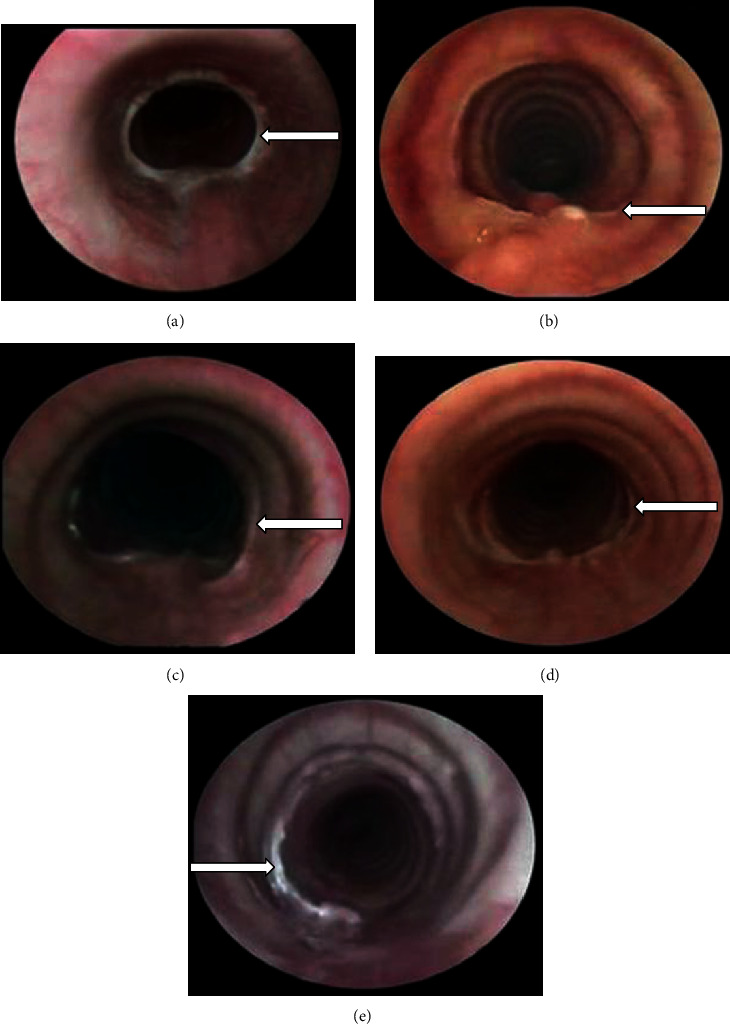
Representative endoscopic view of one animal in each group showing the scar formed in the tracheal anastomosis 4 weeks after treatment. (a) Group I (control): tracheal scar with a concentric membrane of fibrous tissue, which caused grade II stenosis (arrow). (b) Group II (hyaluronic acid), (c) group III (collagen-PVP), and (d) group IV (mixture of hyaluronic acid+collagen-PVP) showing anastomosis scars (arrow) without inflammation or oedema and with 100% patency. (e) Group V (mitomycin C): mild granulation tissue over the mucosa of the tracheal anastomosis (arrow) and 100% of the tracheal lumen.

Tracheoscopically, all animals presented complete tracheal healing on postsurgery day 7. All animals in groups I and V showed inflammation that was characterized by hyperaemia and oedematous mucosa during weeks 1–2, while the dogs in the other groups presented these findings only during week 1 (*p* < 0.05, Kruskal–Wallis). Two animals (33%) in group I developed grade II TS (*p* < 0.05, Kruskal–Wallis) at postoperative week 3, which persisted until the end of the study. Only one dog (16.6%) in group II developed grade I stenosis but showed involution at postoperative week 3 (Figure 1(a)). None of the animals in group III, IV, or V developed TS during the study period (Figures 1(b)–1(d)). However, two animals in group V presented granulation tissue in the scar at the end of the study.

None of the animals presented fistulas, dehiscence, or infection. All animals in group I developed dense and firm fibrous tissue in the outer portion of the trachea and in the mucosa (*p* < 0.05, Kruskal–Wallis); in contrast, the animals of other groups developed mild loose fibrous tissue. All animals in groups I, II, III, and IV showed moderate neovascularization in the tracheal scar, while the animals in group V had mild revascularization (*p* < 0.05, Kruskal–Wallis). On palpation, all tracheal anastomoses treated with HA+collagen-PVP (group IV) showed firm consistency and reduced elasticity.

### 3.2. Inflammation and Fibrosis Microscopically

**Figure 2:**
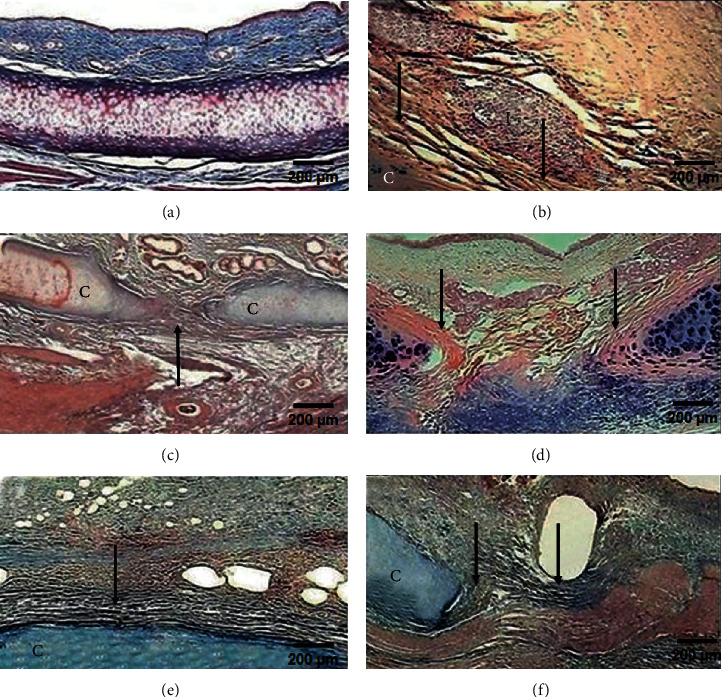
Microphotographs of tracheal scars (Masson's trichrome staining, 2x magnification). (a) Normal histology of the trachea. (b) Group I (control): severe inflammation (I) and disorganized collagen fibres (arrow) around the cartilage (C). (c) Group II (hyaluronic acid), (d) group III (collagen-PVP), (e) group IV (mixture of hyaluronic acid+collagen-PVP), and (f) group V (mitomycin C): well-organized collagen fibres (arrow), mild-to-moderate inflammatory infiltration, and neoformation vessels.

All presurgery tracheal rings were normal in appearance; however, at the end of the study, all groups presented cartilage degeneration and loss of some areas of the tracheal epithelium at the suture line. All animals in group I (*p* < 0.05, Kruskal–Wallis) and one animal (16.6%) in group II showed severe fibrosis (Figures 2(b) and 2(c)). The remaining five dogs (83.4%) in group II and all animals in groups III, IV, and V developed moderate fibrosis (Figures 2(d)–2(f)). In groups I and III, all cases showed new, thick collagen fibres (*p* < 0.001, Kruskal–Wallis), while all the animals in groups II, IV, and V developed thin, well-organized collagen fibres; only group I exhibited disorganized collagen fibres (*p* < 0.05, Kruskal–Wallis). Group V developed mild neovascularization (*p* < 0.001, Kruskal–Wallis), while the other groups showed moderate neovascularization. In group I, the inflammatory reaction was severe in four animals (66.6%) and moderate in two animals (33.4%). In group II, inflammation was moderate in five animals (83.4%) and mild in one animal (16.6%). All animals in groups III and IV presented mild inflammation (*p* < 0.05, Kruskal–Wallis), and group V presented moderate inflammation. Lymphocytes were the main inflammatory cells observed in all layers of the trachea.

### 3.3. Collagen Deposition

**Figure 3:**
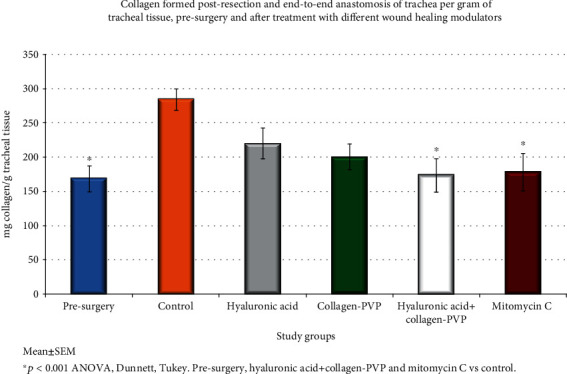
Collagen deposition in tracheal scars prior to surgery and at the end of the study. Each bar represents the mean ± SEM of the collagen concentration in the tracheal tissue for each study group; ^∗^*p* < 0.01 (ANOVA and Tukey's test). The reduced collagen depositions observed in the mixture of hyaluronic acid+collagen-PVP and mitomycin C groups vs. the control group are shown. Group I = control; group II = hyaluronic acid; group III = collagen-PVP; group IV = mixture of hyaluronic acid+collagen-PVP; group V = mitomycin C.

Biochemically, groups IV and V developed a similar degree of collagen deposition to that of the control rings and less than that observed for groups I, II, and III; however, the difference was significant only compared with group I (*p* < 0.001, ANOVA, Dunnett's and Tukey's tests) (Figure 3).

### 3.4. Expression of Decorin

**Figure 4:**
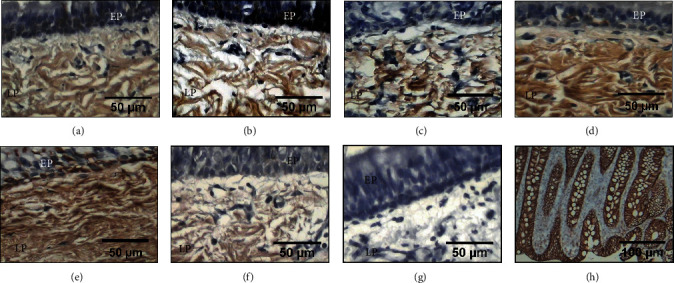
Immunohistochemical detection of decorin in the epithelium (EP) and lamina propria (LP) from tracheal scars presurgery (a) and after treatment with WHMs: (b) group I (control), (c) group II (hyaluronic acid), and (f) group V (mitomycin C) showing light brown immunostaining. (d) Group III (collagen-PVP) and (e) group IV (mixture of hyaluronic acid+collagen-PVP) showing strong brown immunostaining. (g) Tracheal tissue as a negative control. (h) The human colon was the positive control.

**Figure 5:**
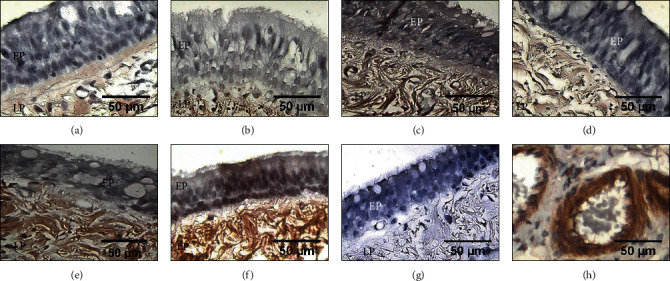
Immunohistochemical detection of MMP1 in the epithelium (EP) and lamina propria (LP) from tracheal scars posttreatment with WHMs. Immunostaining in light brown showing the expression of MMP1 in (a) presurgery samples and in (c) group II (hyaluronic acid), (d) group III (collagen-PVP), and (e) group IV (mixture of hyaluronic acid+collagen-PVP). Strong brown immunostaining indicates MMP1 expression in (b) group I (control) and (f) group V (mitomycin C). (g) Tracheal tissue as a negative control. (h) The vascular endothelium of rat lung tissue was the positive control.

The healed tracheal anastomoses in groups II, III, and IV showed increased expression levels of decorin compared with those in presurgery rings (*p* < 0.01, ANOVA, Dunnett's test) and groups I and V (*p* < 0.01, ANOVA, Tukey's test) (Figures 4 and 5). However, only the expression levels in groups III and IV were significantly different compared with those in groups I and V (*p* < 0.01, ANOVA, Tukey's test) (Table 1 and Figure 4). In all groups, the expression was observed in the epithelium and submucosa.

### 3.5. Expression of MMP1

MMP1 expression in the tracheal epithelium and lamina propria increased at the end of the study in all groups compared with the presurgery levels. In groups II, III, and IV, the increase was moderate; in groups I and V, this increase was marked. Only the levels in groups I and V were significantly different compared with those obtained in presurgery samples and in group II (*p* < 0.003, ANOVA, Dunnett's and Tukey's tests) (Table 1 and Figure 5).

### 3.6. Expression of MMP9

**Figure 6:**
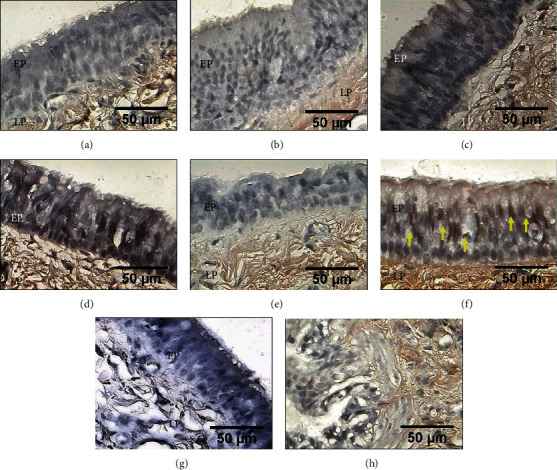
Immunohistochemical detection of MMP9 in the epithelium (EP) (yellow arrows indicate ciliated cells), as well as in tissue of the lamina propria (LP) from tracheal scars posttreatment with WHMs. Light brown immunostaining shows the mild expression of MMP9. (a) Presurgery tissue, (b) group I (control), (c) group II (hyaluronic acid), (d) group III (collagen-PVP), (e) group IV (mixture of hyaluronic acid+collagen-PVP), and (f) group V (mitomycin C). (g) Tracheal tissue as a negative control. (h) The canine lung was the positive control.

In all groups, the epithelium and submucosa of the tracheal scar showed mild increase in the expression of MMP9 compared with that measured in the presurgery samples; however, this difference was not significant (*p* = 0.067, ANOVA), although the expression in group V was increased (Table 1 and Figure 6).

### 3.7. Expression of Different Types of Collagen

**Figure 7:**
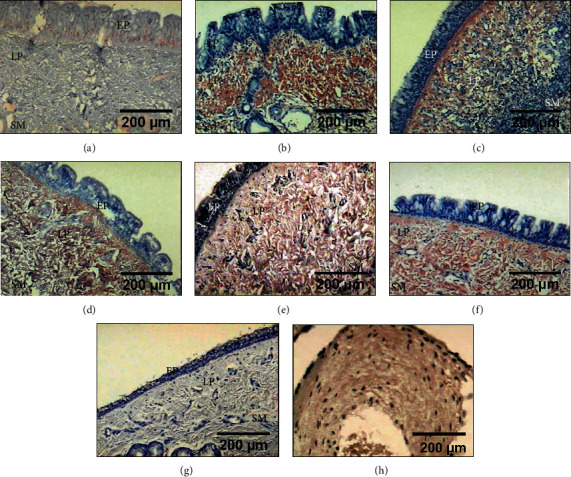
Immunohistochemical detection of collagen type I in the lamina propria (LP) and submucosa (SM) from tracheal tissue, in which severe expression is observed as strong red immunostaining in (b) group I (control) and (f) group V (mitomycin C), as well as moderate expression as light red in the animals of (c) group II (hyaluronic acid), (d) group III (collagen-PVP), and (e) group IV (mixture of hyaluronic acid+collagen-PVP). (a) Presurgery samples. (g) Tracheal tissue as a negative control. (h) The human placenta was the positive control.

In all presurgery rings, the expression of collagen type I was mild, while at the end of the study, the expression was moderate in groups II, III, and IV and severe in groups I and V (*p* < 0.05, Kruskal–Wallis) (Figure 7). In all animals, the expression was observed in the epithelium and lamina propria.

**Figure 8:**
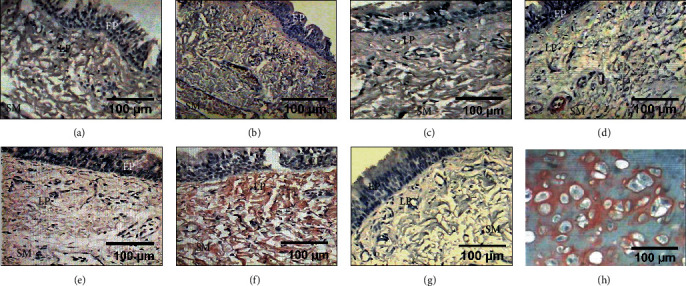
Immunohistochemical detection of collagen type II in the tracheal scars posttreatment with WHMs. Light red immunostaining shows mild expression in all groups: (a) presurgery samples; (b) group I (control); (c) group II (hyaluronic acid); (d) group III (collagen-PVP); (e) group IV (mixture of hyaluronic acid+collagen-PVP); (f) group V (mitomycin C). (g) Tracheal tissue as a negative control. (h) The canine matrix producing carcinoma of the breast was the positive control.

In all cases, both presurgery and at the end of the study, the expression of collagen type II in the cartilage was mild (*p* > 0.05, Kruskal–Wallis) (Figure 8).

**Figure 9:**
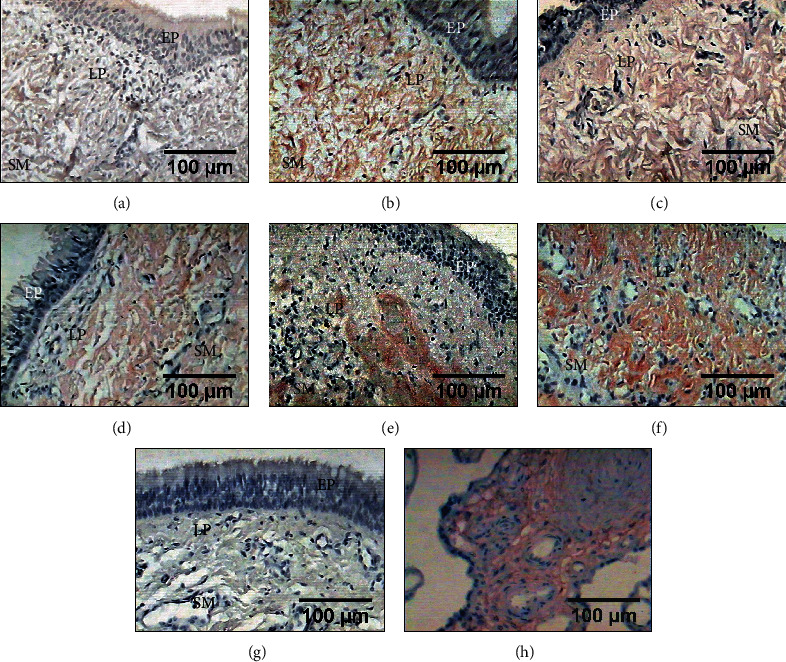
Immunohistochemical detection of collagen type III in the lamina propria (LP) and submucosa (SM) from tracheal tissue. (b) Group I (control) and (f) group V (mitomycin C) showing immunostaining in strong red of the expression of collagen type III. (c) Group II (hyaluronic acid), (d) group III (collagen-PVP), and (e) group IV (mixture of hyaluronic acid+collagen-PVP) showing light red immunostaining which indicates the expression of collagen type III. (a) Presurgery samples. (g) Tracheal tissue as a negative control. (h) The human placenta was the positive control.

Prior to surgery, all groups presented mild expression of collagen type III. At the end of the study, groups I and V showed severe expression (*p* < 0.05, Kruskal–Wallis); however, groups II, III, and IV showed moderate expression. All animals showed expression in the epithelium, lamina propria, and submucosa (Figure 9).

## 4. Discussion

TS results in significant morbidity and can rapidly progress to life-threatening airway compromise. Some clinicians have treated TS in combination with the application of WHM [1, 5] to pharmacologically manipulate the formation of new ECM and prevent fibrosis. However, this approach has not been 100% effective, since it can also cause mucosal damage, inflammation, development of granulation tissue, and restenosis. Experimentally, it has been observed that HA and collagen-PVP allow tracheal healing with less fibrosis [6, 8]. Nevertheless, the effect of these WHMs on ECM components that promote the formation of fibrotic scars has not been described. This knowledge could help investigators understand the reasons these WHMs improve healing in this organ and may provide another clinical treatment option. Therefore, the objective of this study was to evaluate the effect of HA, collagen-PVP, HA+collagen-PVP, and MMC on the expression of decorin, MMP1, and MMP9, as well as the deposition and type of collagen formed in the scar after REEA of CT using an experimental model.

Reduced inflammation and oedema were observed tracheoscopically, macroscopically, and microscopically in the HA, collagen-PVP, and HA+collagen-PVP groups. The reduction of inflammation possibly occurred because HA binds to fibrinogen and controls the recruitment of inflammatory cells, the levels of inflammatory cytokines, and cellular migration [23]. The low oedema was due to the ability of the HA to absorb water molecules from the intercellular space [24]. Moreover, the decrease in the production of proinflammatory cytokines (IL1*β*, TNF*α*, IL17A, and IL22) and the inflammatory volume [7, 25] was due to the effect of collagen-PVP. In contrast, the increased inflammation and delay in its disappearance in the control and MMC groups occurred because there was no anti-inflammatory effect. Furthermore, another study has shown that MMC produces irritation and crusting at the operation site, as well as necrosis of the exposed tracheal cartilage [1]. Similarly, the presence of oedema in the tracheal mucosa could be attributed to reduced reepithelialization and endothelial swelling caused by MMC, which is consistent with intracellular and intercellular vacuole formation and increased vascular permeability [26]. On the other hand, firm consistency of the anastomosis site was detected on palpation at the end of the study in animals in group IV. This was likely due to WHMs altering the production of fibronectin and elastin, causing rigidity and reduced elasticity of the tracheal wall [27]. However, to date, there have been no studies describing this in tracheal healing.

Decorin was observed in the epithelium and submucosa in all of the tracheal healings, since this proteoglycan is produced in these sites throughout the process of scarring of the airway [28, 29]. We did not find any report in the literature regarding the effect of HA, collagen-PVP, and their combination on the expression of this proteoglycan on tracheal healing. However, the results of this study suggest that these WHMs induce decorin expression during tracheal healing, which may explain why both drugs resulted in less fibrosis and collagen deposits. Decorin regulates collagen assembly and its deposition in the ECM during the remodelling phase of wound healing [30]; in addition, it binds to TGF*β*1 and inhibits its activity [24]. Reportedly, collagen-PVP decreases the expression of TGF*β*1 and fibrosis [8]; however, the mechanism of this effect has not been described. As observed in the present study, this phenomenon could have occurred because the drug promotes an increase in the expression of decorin. Conversely, the low expression of decorin observed in the control animals and those treated with MMC was due to the increased inflammation, which decreased the expression of decorin [31].

Well-organized collagen fibres developed in all animals treated with WHMs. This is because, during tissue formation, decorin is inserted and binds to two parallel and contiguous collagen molecules, acting as a spacer to avoid the interaction and lateral fusion among fibres, which favours the balance of distance between them [32]. Similarly, the thin collagen fibres observed in the HA, HA+collagen-PVP, and MMC groups were produced because this proteoglycan decreases the diameter of the fibril in the early stages of fibrillogenesis and promotes fusion, maturation, and thickening of the fibrils in the later stages [33]. These effects suggest that application of collagen-PVP alone promotes the maturation of collagen fibres in less time. Nonetheless, there is no study in the literature that describes this process.

The moderate neovascularization observed macroscopically and microscopically in the groups treated with HA and HA+collagen-PVP occurred as HA binds to CD44. The receptor for cell motility is mediated by HA, which allows the proliferation and migration of endothelial cells and neovascularization [23]. There have been no reports regarding the neovascularization observed in the collagen-PVP group. However, the results of this study indicate that these animals expressed an increased amount of decorin, which promotes motility, endothelial cell germination, cell-matrix interactions, and lumen formation of blood vessels during angiogenesis [34]. In contrast, the low neovascularization shown in animals treated with MMC was due to MMC-mediated inhibition of angiogenesis by blocking the expression of vascular endothelial growth factor [35], as well as inhibiting the growth and inducing apoptosis of microvascular endothelial cells [36].

The presence of MMP1 in the epithelium of all animals was due to the fact that this is a protease produced by airway epithelial cells in several diseases. In airway epithelial repair, the levels of MMP1 are increased during inflammation to degrade components of the injured ECM [3, 37–39]. In the HA, collagen-PVP, and HA+collagen-PVP groups, MMP1 expression was moderate because both drugs decreased inflammation and increased the production of tissue inhibitor of metalloproteinase 1 (TIMP1) [40, 41]. In the MMC group, high expression was observed as topical application causes irritation and prolongs inflammation, consequently leading to overexpression of this MMP [1, 5, 42]. On the other hand, this finding may also be attributed to the lack of MMP1 expression following the complete healing of the tissue [43]. In this study, the healing process in the animals that underwent surgery had not been concluded due to the evaluation time.

The increased production of MMP9 in the epithelium and submucosa in all groups may have occurred because this gelatinase is expressed following injury of the epithelium (tracheal, lung, corneal, or skin). As described by Mohan et al. [4] and Malavia et al. [44] after studying the effect of MMP9 on epithelial regeneration and wound healing, its function during the healing process is to enable cell migration through the ECM to reepithelialize the wounded area. However, the increased expression of MMP9 in the animals treated with MMC occurred because MMC exerts proinflammatory effects that stimulate the expression of different MMPs [42].

All tracheas after surgery showed expression of collagen types I and III in the epithelium, lamina propria, and submucosa, since the remodelling process of airways begins in these areas where both collagen types are involved [45]. Moreover, all animals expressed less type III collagen than type I collagen posttreatment with different WHMs. This is because these factors stimulate adequate tracheal healing, as observed in other organs in which this process begins with an increase in the expression of type III collagen (as part of granulation tissue), which is replaced after week 3 by type I collagen (as part of the new scar tissue) [43, 46]. Our results suggest that the decrease in inflammation and production of growth factors, as well as the increase in decorin expression and regulation of MMP1 expression induced by HA, collagen-PVP, and HA+collagen-PVP, promotes a balance in the expression of these types of collagen [7, 41, 47, 48]. Nevertheless, as reported by Choi et al., the increased expression of both types of collagen in the MMC group was due to drug-induced inflammation and activation of division of myofibroblasts and fibroblasts, causing an immediate increase in collagen production, mainly collagen type I [49].

In all cases, the expression of collagen type II observed in cartilage was due to the fact that it is produced in the ECM of all cartilage [50]. Furthermore, the mild presence of type II collagen in all animals presurgery and at the end of the study occurred because type II collagen is less susceptible to inflammation and has the ability to regenerate in the injured trachea [51]. Some studies have reported that the application of HA [52] or collagen-PVP [53] in articular cartilage promotes the expression of this collagen, which is not consistent with the observations of this study. This finding suggests that the application of these drugs does not increase the expression of type II collagen in injured tracheal cartilage. Additionally, there have been no reports regarding the effect of MMC on the expression of type II collagen during tracheal healing. However, based on the observations of this study, it is possible that MMC does not affect the expression of type II collagen during tracheal healing.

## 5. Conclusion

Treatment with HA and collagen-PVP improved post-REEA healing by increasing neovascularization, favouring the expression of decorin, and regulating the expression of MMP1, as well as type I and III collagen and their deposition. Although these results provide evidence on the mechanism through which tracheal fibrosis is decreased, more experimental studies are warranted to evaluate their effect on animals with TS. Similarly, another form of drug administration is needed, because it is not feasible to apply weekly endotracheal treatments for a month. We also think that it is necessary to design a clinical trial that proves the usefulness of this treatment in airway surgery.

## Figures and Tables

**Table 1:** Expression in pixels of the different ECM proteins in all study groups prior to surgery and at the end of the study. Values are expressed as the mean ± SEM of pixels quantified in the entire sample circumference. Group I = control; group II = hyaluronic acid; group III = collagen-PVP; group IV = mixture of hyaluronic acid+collagen-PVP; group V = mitomycin C.

Study groups	Decorin	MMP1	MMP9
Presurgery	137118 ± 23010	136610 ± 28434	292501 ± 69240
Group I (control)	164822 ± 39279	253198 ± 47942	412707 ± 107480
Group II (hyaluronic acid)	172117 ± 23980	345276 ± 63589	368170 ± 98770
Group III (collagen-PVP)	188993 ± 15640^∗^	410434 ± 63943	354796 ± 102972
Group IV (hyaluronic acid+collagen-PVP)	195018 ± 18700^∗^	412962 ± 68986	351743 ± 71051
Group V (mitomycin C)	156037 ± 25110	619117 ± 32243^∗∗^	379062 ± 127044

Expression based on pixel analysis of decorin, MMP1, and MMP9 postresection and end-to-end anastomosis of trachea, presurgery, and after treatment with different wound healing modulators. Mean ± SEM. ^∗^*p* < 0.01: ANOVA, Dunnett, and Tukey. Groups III and IV vs. presurgery and groups I and V. ^∗∗^*p* < 0.003: ANOVA, Dunnett, and Tukey. Group V vs. presurgery and groups II and III.

## Data Availability

The data used to support the findings of this study are included within the article.
